# Quality of Life in Women with Coronary Artery Disease

**DOI:** 10.5812/ircmj.10188

**Published:** 2014-07-05

**Authors:** Elham Ghasemi, Jaleh Mohammad Aliha, Farideh Bastani, Hamid Haghani, Niloufar Samiei

**Affiliations:** 1 Nursing and Midwifery Care Research Center, Tehran University of Medical Sciences, Tehran, IR Iran; 2 Department of Critical Care Nursing, School of Nursing and Midwifery, Iran University of Medical Sciences, Tehran, IR Iran; 3 Department of Geriatric Nursing, School of Nursing and Midwifery, Iran University of Medical Sciences, Tehran, IR Iran; 4 School of Health Management and Information Sciences, Iran University of Medical Sciences, Tehran, IR Iran; 5 Heart Valve Disease Research Center, Rajaei Cardiovascular Medical Research Center, Iran University of Medical Sciences, Tehran, IR Iran

**Keywords:** Quality of Life, Coronary Artery Disease, Women

## Abstract

**Background:**

Coronary artery disease (CAD) as a chronic disease can affect physical, mental, and social aspects of health as well as the perception of wellbeing. Advanced treatments of the disease emphasize on functionality and quality of life (QOL).

**Objectives:**

The present study aimed to investigate the QOL and its related factors among women with CAD.

**Patients and Methods:**

This was a descriptive cross-sectional study conducted on 200 women with CAD, referring to the Heart Clinic of Shahid Rajaei Cardiovascular Center in Tehran, Iran. The participants were selected by convenient sampling method. Data were collected using the Persian version of Ferrans and Powers QOL index (QLI) cardiac version and then analyzed using descriptive statistics and statistical tests (independent t-test, one-way ANOVA, and Scheffe’s test).

**Results:**

The mean score of overall QOL was 16.91 ± 3.54, ranging between 7.17-27.63. Regarding the instrument subscales, the mean scores were as follows: health and functioning: 15.48 ± 4.32, social and economic: 16.18 ± 3.65, psychological/spiritual: 18.04 ± 4.36, and familial: 20.12 ± 4.57. There was a significant relationship between QOL and marital status (P = 0.004), education (P = 0.007), income (P < 0.001) and disease duration (P = 0.047). However, there was no significant association between QOL and age, job and comorbidity.

**Conclusions:**

Based on the findings, participants had average levels of overall QOL. Some domains showed the need to improve QOL of women with CAD. Results of the present study revealed the necessity of designing and performing educational and supportive interventions to improve the QOL in women with CAD, especially among patients with low socio-economic status.

## 1. Background

Coronary artery disease (CAD) is the most common form of heart disease (1) and the leading cause of death worldwide. Among diseases, it is the most prevalent cause of death in women (2), leading to death of 0.5 million women per year in the United States (3). Three-fourth of deaths due to CAD occur in the low and middle-income countries. According to the evidence, Iran has a higher burden of CAD than some countries in the Middle East and North Africa including Saudi Arabia and Jordan (4). Based on several epidemiological studies in Iran, the prevalence of CAD among women is more than men, revealing that health organizations should pay more attention to women with CAD (5, 6). Previously, morbidity and mortality rates were measured as outcomes of CAD, but nowadays, there is more attention to quality of life (QOL) as an important indicator of effectiveness of interventions (2). QOL is defined by Ferrans as "a person's sense of well-being that stems from satisfaction or dissatisfaction with the areas of life that are important to him/her" (7). CAD, as a chronic disease, can affect physical, mental, and social aspects of health as well as individual’s perception of wellbeing and advanced treatments of CAD emphasize on functionality and QOL (8-10). Patients with CAD are mostly worried about worsening of symptom and physical functions as well as changes in their social roles due to disease progression (10). Patients usually have some problems including physical dysfunction, sleep disturbances, low energy, and emotional reactions. Depending on the disease severity, various levels of QOL are experienced by people (11). Low QOL in people with heart disease is associated with an increasing rate of rehospitalization and cardiac death (12). According to the evidence, women with CAD have lower general and mental health conditions, physical and social functioning, and QOL compared with men (2). According to the study conducted by Norris et al. one of the factors affecting the differences of QOL between men and women with CAD is the defined roles for each gender in the society (13). Multiple roles of women as spouse, mother, housewife and probably employee can be among causes of ignoring their health-related issues. In addition, most women consider others’ needs superior to theirs, which is mostly seen in Iranian women’s culture. Therefore, it is very important to measure their senses of well-being. There is consensus that QOL is a subjective and multidimensional concept comprised of physical, functional, social, emotional, and mental domain and it is important to measure the dimensions of health, particularly in people with specific diseases (11, 14).

## 2. Objectives

Regarding the lack of research on QOL in this group of patients in Iran, this study was conducted to determine the QOL in women with CAD considering a variety of domains, using a disease-specific QOL instrument, as well as factors associated with QOL in these patients.

## 3. Patients and Methods

A descriptive-analytic cross-sectional design was used in this study. Two hundred women with CAD who referred to the outpatient clinics of Shahid Rajaei Cardiovascular Medical and Research Center in Tehran, Iran were selected, using convenience sampling method from July to October 2010. This center is one of the largest governmental hospitals in Middle East. The center currently features a total of 601 beds, providing medical services for patients with cardiovascular disease from all parts of Iran and neighboring countries. Patients, aged 18 and older, who dwelled in Tehran and according to their medical records were diagnosed with stable or unstable angina and also who had ejection fraction above 40%, were included. In addition, subjects should not have a history of myocardial infarction, cancer, renal failure, or stroke. Known cases of psychological or cognitive disorders or previous physical limitations in self-care were also excluded. According to the sample size, the study questionnaires were completed by face-to-face interview with 200 eligible women who agreed to participate. Based upon Hagell and Westergren’s study (7) that reported the standard deviation of overall QOL (SD = 4.3), and also considering α = 0.05 and d = 0.6, the sample size was estimated as 200 cases.

n = (z_1_ - α/2)^2^ × SD^2^) / d^2^ = (3.84 × 18.49) / 0.36 = 200

In this study two questionnaires were used: one collected demographic data such as age, marital status, educational level, income, job situation, duration of the disease, and co morbidity; the other one was the Ferrans and Powers QOL index (QLI) cardiac version, which was translated to Persian and used to measure the QOL (15). It is a self-administered measure of QOL, consisted of two parts with 35 items: the first part measures satisfaction with various aspects of life and the second measures importance of those in the same aspects. The participants responded to each item using a 6-point Likert-type scale with end points of very dissatisfied/very unimportant and very satisfied/very important. Scores were calculated for QOL overall and in four domains: health and functioning, social and economic, psychological/spiritual, and family. The possible range for the final score is 0 to 30 and higher scores indicate greater perceived QOL (16). The QLI is a well-established instrument with substantial evidence of reliability and validity, which has been supported in various studies among patients with CAD (17, 18). In a previous study in Iran, validity of this instrument in Persian language was confirmed and its reliability was supported using Cronbach’s alpha of 0.86 (15).

The study was approved for human subjects’ protection by the institutional review board of Iran University of Medical Sciences as well as the Nursing Research Committee of the university (Code: 1676385, Date: 24/05/2010). Written informed consents were obtained from all patients prior to inclusion in the study. An introductory letter was provided to the potential participants, containing information about the procedure, sharing the purpose of the study and study results, to assure the anonymity in publication of the study results. The patients were free to quit the study at any time. They were also assured that participation/nonparticipation would not affect the care they received.

Data were analyzed using SPSS version 14 for Windows. Descriptive statistics (frequency distribution percentage, mean and standard deviation) were carried out to describe demographic and clinical data. To test the distribution normality of the QOL data, Kolmogorov-Smirnov test was performed. Independent t-test and one-way ANOVA were used to assess the relationship between overall QOL and independent variables. In cases that ANOVA results were statistically significant, Scheffe’s test was used to provide specific information, in which the mean values were significantly different from each other. P value less than 0.05 was considered significant.

## 4. Results

The mean age of subjects was 62.51 ± 9.48, ranging from 39 to 87 years. Most of the subjects were married (68%), 48.5% were illiterate or had no educational qualification, 57% perceived their income as average, and the majority were housewives (97%). Moreover, comorbidity was also frequent (96%). Duration of the disease ranged 1-47 year with a mean of 9.39 ± 8.68. Table 1 shows the demographic characteristics of patients (Table 1).

The mean score of overall QOL was 16.91 ± 3.54, ranging between 7.17-27.63. With respect to the domains of QOL, mean and standard deviation scores were health and functioning: 15.48 ± 4.32, social and economic: 16.18 ± 3.65, psychological/spiritual: 18.04 ± 4.36, and familial subscale: 20.12 ± 4.57 (Table 2).

Data analysis revealed significant relationship between QOL and marital status, educational level, income and disease duration (P < 0.05). According to the Scheffe’s test, married patients had higher QOLs compared with unmarried ones. In addition, patients with high school grades or higher educational levels had higher QOLs than illiterate patients. The patients with average or good income had higher QOLs compared with ones with low economical statuses. There was no significant association between QOL and other demographic variables (Table 3).

**Table 1.** Demographic Characteristics of the Participants (n = 200)

Characteristics	Frequency, No. (%)
**Age, y**	
50 >	19 (9.5)
50-60	47 (23.5)
60-70	84 (42)
70 ≤	50 (25)
**Marital status**	
Unmarried	64 (32)
Married	136 (68)
**Educational status**	
Illiterate	97 (48.5)
Primary school	69 (34.5)
Middle school	18 (9)
High school and higher	16 (8)
**Economic status**	
Low	79 (39.5)
Average	114 (57)
Good	7 (3.5)
**Had job**	
Yes	6 (3)
No	194 (97)
**Disease duration, y**	
5 >	68 (34)
5-10	52 (26)
10-15	40 (20)
15-20	14 (7)
20 ≤	26 (13)
**Comorbidity**	
Yes	192 (96)
No	8 (4)

**Table 2.** Description of Quality of Life According to Dimensions of Quality of Life Index and Overall Quality of Life

Variables	Mean ± SD	Ranges of Scores
**Overall quality of life**	16.91 ± 3.54	7-27
**Health and functional status**	15.48 ± 4.32	5-28
**Social and economic status**	16.18 ± 3.65	7-32
**Psychosocial/spiritual status**	18.04 ± 4.36	8-30
**Family status**	20.12 ± 4.57	4-29

**Table 3.** The Association Between Demographic Variables of Participants and Overall Quality of Life

Demographic Variables	Overall Quality of Life, Mean ± SD	P Value
**Age**		0.233
50 >	17.66 ± 2.35	
50-60	17.13 ± 3.77	
60-70	17.13 ± 3.90	
70 ≤	16.05 ± 2.97	
**Marital status**		0.004
Unmarried	15.86 ± 3.61	
Married	17.41 ± 3.41	
**Educational status**		0.007
Illiterate	16.31 ± 3.47	
Primary school	16.93 ± 3.13	
Middle school	17.97 ± 3.28	
High school and higher	19.32 ± 4.81	
**Economic status**		< 0.001
Low	15.41 ± 3.50	
Average/Good	17.90 ± 3.22	
**Had job**		0.108
Yes	19.21 ± 2.24	
No	16.84 ± 3.51	
**Disease duration, y**		0.047
5 >	17.04 ± 3.94	
5-10	17.13 ± 2.70	
10-15	17.85 ± 3.76	
15-20	15.45 ± 4.14	
20 ≤	15.50 ± 2.77	
**Comorbidity**		0.549
Yes	16.88 ± 3.51	
No	17.65 ± 4.37	

## 5. Discussion

Based on the findings, participants had average levels of QOL. These findings showed that QOL in women with CAD needs improvement. These findings are important because CAD has high prevalence among women (5, 6). On the other hand, women comprise half of the world population and play significant roles in families. In fact, family health depends on woman’s health. Previous studies reported that in comparison to the general population, patients with CAD had lower QOLs (19, 20). Our finding was supported by Durmaz et al. study; they reported that the average level of overall QOL score measured by QLI in patients with CAD was 16.0 ± 0.94 (16). Penckofer et al. (17) reported higher QOL scores in women before CABG (Coronary Artery Bypass Grafting) compared with our findings. Norekval et al. reported that 67% of female MI (Myocardial Infraction) survivors had good or very good overall QOLs, which was 79% in the general female population group, while only 6% rated their overall QOL as low or very low, compared with 4.5% in the general female population (20). Therefore, our participants showed similar or lower levels of QOL, showing that health care planners should consider this difference as a priority in improvement of QOL among women with CAD and without the history of MI. According to the findings, married women with better incomes and higher educational levels had higher overall scores of QOL. It is accepted that married people are more supported by families than singles, which has a considerable role in coping with diseases, resulting in better QOL scores. Luttik et al. believed that supportive resources are necessary for survival and adaptation to the disease and cardiac patients who live alone have low QOLs (21). Moreover, the individuals with higher economic and educational levels have more access to information, and consequently, use effective strategies for coping with heart disease and thus, have higher QOLs. Similar to these findings, in previous studies, cardiac patients with higher educational levels (17, 22, 23) or better incomes (17) reported higher QOLs.

The strength of this study was measurement of QOL. The QLI, cardiac version, is a standard disease-specific instrument for cardiac patients, which represents various domains related to QOL among patients with CAD. Disease-specific instruments comprise important aspects of health considered by patients or clinicians. These instruments have the potential to make them more responsive to changes in health, together with more detailed and accurate assessment (24). We studied women with stable and unstable angina who did not have any history of MI; thus, we controlled the effect of MI on QOL in these patients.

There were some limitations in this study that could influence the generalizability of findings. Recruitment of sample was conducted in one center; however, Shahid Rajaei Cardiovascular Medical and Research Center is one of the largest heart hospitals and many cardiac patients are referred to it; nonetheless, it is recommended that similar studies should be performed in multicenter settings. Furthermore, nonrandomized convenience sampling was used due to time limitation and easy access to patients; so, a study with randomized sampling method is recommended.

Based on the findings, QOL in women needs improvement and various factors that affect QOL should be considered. Nurses need to be knowledgeable regarding QOL in women with CAD, be able to identify persons at risk, and provide appropriate patient education and psychosocial support on how to improve several aspects of QOL. The main aim of treatments and care of patients with coronary diseases is improving their physical, mental and social functionality. Findings of the study emphasized on the necessity of improving QOL in women with CAD, especially in low socio-economic status groups. These results recommend the treatment planners to have a holistic view in assessment of these patients and consider all domains of health and functioning, socio-economic status, and psychological-spiritual and familial QOL. They should refer patients to supportive resources and plan effective educational and supportive interventions with multidisciplinary approach, aimed to improve QOL. Furthermore, it is valuable to examine the changes of QOL among these patients over the time.
